# DNA Barcoding Bromeliaceae: Achievements and Pitfalls

**DOI:** 10.1371/journal.pone.0029877

**Published:** 2012-01-05

**Authors:** Vitor Hugo Maia, Camila Souza da Mata, Luciana Ozório Franco, Mônica Aires Cardoso, Sérgio Ricardo Sodré Cardoso, Adriana Silva Hemerly, Paulo Cavalcanti Gomes Ferreira

**Affiliations:** 1 Instituto de Pesquisas Jardim Botânico do Rio de Janeiro, Diretoria de Pesquisa Científica, Rio de Janeiro, Rio de Janeiro, Brazil; 2 Instituto de Bioquímica Médica, Universidade Federal do Rio de Janeiro, Rio de Janeiro, Rio de Janeiro, Brazil; Biodiversity Insitute of Ontario - University of Guelph, Canada

## Abstract

**Background:**

DNA barcoding has been successfully established in animals as a tool for organismal identification and taxonomic clarification. Slower nucleotide substitution rates in plant genomes have made the selection of a DNA barcode for land plants a much more difficult task. The Plant Working Group of the Consortium for the Barcode of Life (CBOL) recommended the two-marker combination *rbcL*/*matK* as a pragmatic solution to a complex trade-off between universality, sequence quality, discrimination, and cost.

**Methodology/Principal Findings:**

It is expected that a system based on any one, or a small number of plastid genes will fail within certain taxonomic groups with low amounts of plastid variation, while performing well in others. We tested the effectiveness of the proposed CBOL Plant Working Group barcoding *markers* for land plants in identifying 46 bromeliad species, a group rich in endemic species from the endangered Brazilian Atlantic Rainforest. Although we obtained high quality sequences with the suggested primers, species discrimination in our data set was only 43.48%. Addition of a third marker, *trnH–psbA*, did not show significant improvement. This species identification failure in Bromeliaceaecould also be seen in the analysis of the GenBank's *matK* data set. Bromeliaceae's sequence divergence was almost three times lower than the observed for Asteraceae and Orchidaceae. This low variation rate also resulted in poorly resolved tree topologies. Among the three Bromeliaceae subfamilies sampled, Tillandsioideae was the only one recovered as a monophyletic group with high bootstrap value (98.6%). Species paraphyly was a common feature in our sampling.

**Conclusions/Significance:**

Our results show that although DNA barcoding is an important tool for biodiversity assessment, it tends to fail in taxonomy complicated and recently diverged plant groups, such as Bromeliaceae. Additional research might be needed to develop markers capable to discriminate species in these complex botanical groups.

## Introduction

Taxonomy is a key science to understand and monitor biodiversity. However, since taxonomists first started classifying organisms, roughly 1.7 million species have been described, representing only a small fraction of the estimated tens of millions extant species on Earth [1]. This descriptive deficit varies widely, not only among different taxa but also between countries. Unfortunately, the richest inbiodiversity are the poorest in human and material resources. Moreover, the tropics, which harbor the majority of Earth's species, are potentially threatened by global climate change [2], [3]. Human activities are also causing the extinction of species hundreds of times faster than the natural rate of extinction found in the fossil record [1]. With the predicted climate change and no immediate change in human behavior, the biosphere will be drastically modified within just a few decades, with the number of species facing extinction being estimated at one million by 2050 [4].

This urgent need to inventory and manage diversity created a demand for innovative tools to identify species, such as DNA barcoding, proposed by Hebert *et al.* [5]. A DNA barcode is a short, highly variable, standardized, orthologous DNA sequence used to identify species through comparison to a DNA sequence database. It has been most successfully applied to animals using the mitochondrial cytochrome c oxidase I gene. The *COI*, or *coxI* gene, is variable enough to allow discrimination among closely related species, and yet possesses highly conserved regions that can be easily sequenced with standard protocols [5], [6].

Because of slower nucleotide substitution rates in plant mitochondrial DNA [7], plastids have become the primary source of DNA barcodes in plants. After evaluating the performance of seven leading candidate plastid DNA regions (*atpF–atpH*, *psbK–psbI*, *trnH–psbA* spacers and *matK*, *rbcL*, *rpoB*, *rpoC1* genes), the Plant Working Group of the Consortium for the Barcode of Life (CBOL) recommended the two-marker combination *rbcL*/*matK* as the standard DNA barcode for plants [8].

It is expected that a system based on any one or small number of plastid genes will fail in certain taxonomic groups with low amounts of plastid variation, while performing well in others [9]. The purpose of the current study is to test the effectiveness of the proposed markers in Bromeliaceae, a group rich in endemic species in the endangered Brazilian Atlantic Rainforest.

The Atlantic Rainforest is a hot spot of plant biodiversity, with a large number of endemic species. Most natural populations of these species are comprised of a low number of individuals and it's believed that for every two endangered Brazilian trees, one is found exclusively in the Atlantic Rainforest ecosystem. Because of its the proximity to urban centers, this complex ecosystem, one of the most threatened in the world, has been the subject of environmental degradation by social and economic pressures. During the last 500 years, the geographical range of the Atlantic Rainforest has been reduced from 12% to 1% of the Brazilian territory.

## Materials and Methods

### Plant Material

A total of 101 individuals, comprising 46 species were sampled from the Dimitri Sucre greenhouse, which holds the Bromeliaceae scientific collection of the Instituto de Pesquisas Jardim Botânico do Rio de Janeiro, in Rio de Janeiro/RJ, Brazil. Most individuals sampled in this study derived from plants collected in scientific expeditions led by Dr. Gustavo Martinelli. Vouchers of each specimen are deposited in the Herbarium (RB) of the Instituto de Pesquisas Jardim Botânico do Rio de Janeiro. Specimens' vouchers and respective GenBank accessions are available in Table S1.

### DNA Extraction, Amplification and Sequencing

Young leaves were collected, cleaned, torn by hand when possible and immediately frozen in liquid nitrogen or dried in silica gel. Frozen tissues were disrupted in liquid nitrogen using a mortar and pestle. Dry material was ground in a mixer-mill disruptor (Mixer Mill 301 Confort, Retsch) using stainless steel beads. Total genomic DNA was isolated using modified CTAB protocol. [10]. All DNA extracted is deposited in the DNA Bank of Brazilian Flora Species, Instituto de Pesquisas Jardim Botânico do Rio de Janeiro.

The two proposed plastid barcoding markers for plants, *matK* and *rbcL* [8], were evaluated using the described sets of primers: 390F/1326R for *matK* [11] and 1F/724R for *rbcL* [12]. The noncoding *trnH-psbA* spacer was also tested using primers psbA3'f/trnHf described by Kress *et al.*, 2005 – PNAS 102 (23): 8369–8374.

PCR reactions contained 25 ng of DNA template, 1× reaction buffer (10×: 10 mM Tris-HCl pH8.5, 50 mM KCl, 1.5 mM MgCl2, 0,01% gelatin), 0,2 mM dNTPs, 10 pmol of each primer and 2.5 units of Taq DNA polymerase, resulting in a final volume of 25 µL. Reactions with *matK* primers also contained 4% of the total reaction volume of DMSO. BSA was added in *rbcL* and *trnH-psbA* amplifications for a final concentration of 0.5 µg/µL.

The following PCR profiles were used:

1. *matK*: 94°C 5 min; [26 cycles: 94°C 1 min; 48°C 30 sec; 72°C 1 min]; 72°C 7 min;
2. *rbcL*: 94°C 5 min; [30 cycles: 94°C 1 min; 50°C 30 sec; 72°C 1 min]; 72°C 7 min;
3. *trnH*-*psbA*: 94°C 5 min; [30 cycles: 94°C 30 sec; 48°C 30 sec; 72°C 30 sec]; 72°C 5 min

PCR products were purified and sequenced at Macrogen Inc., Seoul, South Korea. Sequencing was conducted under BigDye™ Terminator v3.1 cycling conditions. The reacted products were purified using ethanol precipitation and run using an ABI3730XL automatic sequencer.

### Data Acquisition from GenBank

*matK* sequences of Bromeliaceae, Asteraceae and Orchidaceae were downloaded from GenBank. Sequences with ambiguous bases (more than 10′Ns' in the whole sequence), or those belonging to unnamed species (i.e. sequences with ‘sp.’ in the species name) were filtered out. Analyses were conducted with the selected sequences, constituting different data sets for each family (see Table S2 for GenBank accessions). All sequences were equally trimmed in order to make comparisons among data sets possible.

### Data Analysis

Sequences were assembled and edited in Geneious Pro 5.0.4 software (Biomatters Ltd.). Prior to assembling, sequences were trimmed based on the quality values of the traces, using the Modified-Mott algorithm implemented on the software. Contig quality was assessed by Confidence Mean value, which is the mean of confidence scores for the contig base calls.

Sequence alignments were conducted on MUSCLE (version 3.8.31) [13], using default parameters, and subsequently checked by visual inspection. The optimality criterion GLOCSA [14] was applied to evaluate the best alignment option, using the software GLOCSER (downloaded at goten.ibiologia.unam.mx/glocsa/wp-content/uploads/2010/12/glocser-sept2010.zip).

Phylogenetic analyses were made with the combined data sets, using three different methods: maximum parsimony (MP), distance, and maximum likelihood (ML). *Typha angustifolia* sequences downloaded from GenBank (accessions GQ436382, AY952419 and EU750604) were used as an outgroup. Parsimony analyses were performed using PAUP* (version 4.0b10) [15]. Tree searches were conducted using heuristic search option RANDOM addition sequences (1000 replicates) holding 10 trees per replicate, and tree bisection-reconnection (TBR) branch swapping, with retention of multiple parsimonious trees (MAXTREES was set to 5000). Branch support was given by 1000 bootstrap pseudoreplicates, with the following parameters for tree search: simple taxon addition, TBR branch swapping, and holding 10 trees at each step. Incongruence between marker partitions was evaluated by the Incongruence Length Difference (ILD) test [16].

Distance analysis was carried out on MEGA 5 [17], using the NJ algorithm and Jukes-Cantor as the model of substitution. The choice of this model was based on Nei and Kumar [18] (overall mean: p distance = 0.010 transitions/transversions = 2.09). For the ML analysis, HKY+I+G model was selected as the best-fit model by AIC implemented in jModeltest (version 0.0.1) [19]. PHYML (version 3.0) [20] was used for phylogeny estimate with a BIONJ starting tree [21]. Bootstrap support was accessed by 1000 and 100 pseudoreplicates in NJ and ML analyses, respectively.

MEGA5 was also used to calculate K2P pairwise and overall distances to each one of the previously designated DNA barcode regions. In order to compare *matK* variation in different plant families, we also calculated K2P overall distance to the GenBank's data sets.

A local BLAST search method was performed to test species identification capability, as described previously [22]. This method was applied to all data sets (GenBank, *rbcL*, *matK*, *trnH-psbA* and combinations of regions). In short, a reference library was constructed using the “makeblastDB” command in BLAST+ [23] for each region and combination of regions (*rbcL*+*trnH-psbA*, *rbcL*+*matK*, *matK*+*trnH-psbA* and *rbcL*+*matK*+*trnH-psbA*). The barcode sequence of each species was then queried against the reference library with the “blastn” command. Only species matching 100% in sequence similarity over the entire sequence length with their own reference sequence were counted as successful identifications; those that also matched 100% over the entire sequence length but had the reference sequence of one or more different species were counted as failures.

All statistical analyses regarding sequence quality, PCR/sequencing success, and species discrimination among markers were conducted on STATISTICA software (version 7; StatSoft Inc., 2004), using the test of Tukey at P = 0.05.

## Results

### Success rates for PCR/sequencing and Sequence Quality

*matK* sequences were obtained from all 101 DNA samples, while *rbcL* and *trnH-psbA* sequences could only be obtained from 92 and 99 samples, respectively. Polymerase chain reactions (PCR) of the three regions were always successful, but sequencing failed for these missing samples. PCR/sequencing success was statistically different only for *rbcL* (Tukey test; P = 0.05). The average sequence quality values (Confidence Mean scores) were 46.6, 46.5 and 43.1 for *rbcL*, *trnH-psbA* and *matK*, respectively. Although *matK* presented a satisfactory sequence quality, it was significantly different from the other two markers.

### Distance and Phylogenetic Analyses

Sequences from *rbcL* and *matK* data sets resulted in 632 bp and 797 bp length alignments, respectively, with no indels. The *trnH-psbA* aligned matrix was 679 bp long with 29 indels, ranging from 1 to 38 bp (mean size = 11.4). Possible adjustments on *trnH-psbA* alignment were not done since MUSCLE generated the alignment with the best GLOCSA score (815.16). Sequences without complete data set were excluded from combined analysis.

**Figure 1:**
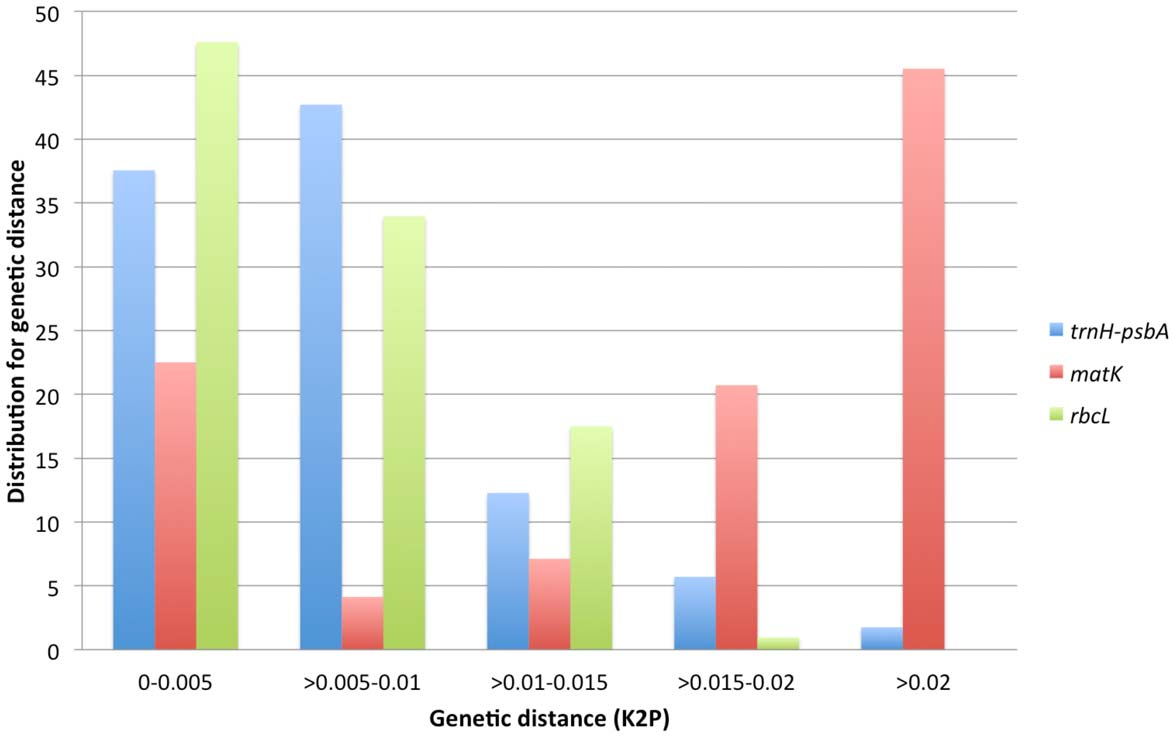
Relative distribution of K2P distances across all sequence pairs of Bromeliaceae data set for different markers.

Figure 1 shows the distribution of interspecific K2P distances among all sequences that were successfully obtained for *matK*, *rbcL* and *trnH-psbA*.

Estimates of average evolutionary divergence over all GenBank's *matK* data set for Bromeliaceae, Asteraceae and Orchidaceae, using K2P model, are shown in the Table 1.

**Table 1:** Comparison of identification efficiency of *matK* region among GenBank's data sets, representing three angiosperm families.

Families	N° Sequences	N° Species	Success Rates (Blast Test)	Overall K2P Distance
Asteraceae	778	577	74.87%	4.8%
Bromeliaceae	422	334	52.40%	1.9%
Orchidaceae	365	297	64.67%	6.2%

### Phylogenetic Analyses

**Figure 2:**
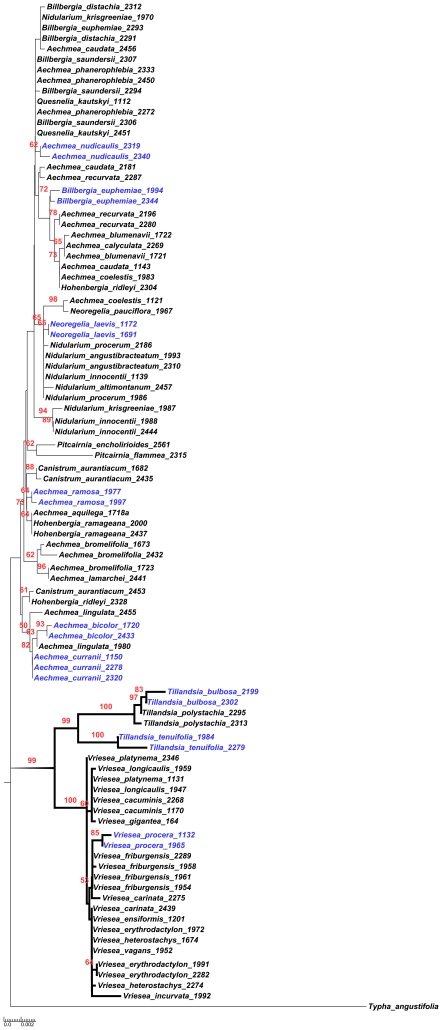
NJ tree of genetic distance (JC) for 91 Bromeliaceae specimens, based on *rbcL*, *matK* and *trnH-psbA* combined analysis. Genera and species recovered as monophyletic are highlighted (thicker branches and names in blue, respectively). Numbers above branches correspond to bootstrap support ≥50%.

For the phylogenetic analyses, the combined data set was used since ILD test showed no significant difference among markers (P = 0.082). All three phylogenetic reconstruction methods recovered very similar and poorly resolved topologies. MP analysis resulted in 5000 equally parsimonious trees (L = 318). The NJ tree is shown in Figure 2. Of the 35 species with multiple accessions, only six were recovered as monophyletic in all analyses.

### Blast Analysis

Summaries of species discrimination rates based on the three regions and their combination are shown in Table 2. The single region with highest success rate is *matK* (27.66%). The combination of the three regions increases success rate to 44.44%. Statistical significance among markers and their combinations is shown in Table S3.

**Table 2:** Comparison of identification efficiency of the plastid regions and their combinations in Bromeliaceae data set.

Region	N° Species (Correct Identification)	Success Rates (Blast Test)	Overall K2P Distance
*rbcL*	47 (9)	19.15%	0.6%
*matK*	47 (13)	27.66%	1.4%
*trnH-psbA*	46 (12)	26.09%	0.7%
*rbcL*+*matK*	46 (20)	43.48%	-
*rbcL*+*trnH-psbA*	45 (12)	26.67%	-
*matK*+*trnH-psbA*	46 (16)	34.78%	-
*rbcL*+*matK*+*trnH-psbA*	45 (20)	44.44%	-

Blast test performed with GenBank's data sets (Table 1) exhibited the lowest discrimination rate for Bromeliaceae (52.40%).

## Discussion

DNA barcoding has helped to rejuvenate taxonomy, a key science to understanding and monitoring biodiversity. Estimates of regional species diversity, abundances, and geographic distributions are essential to comprehend how species-rich communities are assembled and how they might be conserved.

In this study, we tested the effectiveness of the proposed CBOL Plant Working Group barcoding *markers* for land plants in identifying 46 bromeliad species. Bromeliaceae form a large, morphologically distinctive, ecologically diverse and species-rich family of angiosperms, native to the tropics and subtropics of the New World [24]. Many species are endemic to the Atlantic Rainforest, one of the most threatened tropical ecosystems in the world.

The *rbcL*/*matK* two-marker combination was proposed as the standard plant barcode for its universality, sequence quality, discrimination, and costs. Although we obtained high quality sequences with the suggested primers, species discrimination in our data set was 43.48%; much lower than the 72% success found by CBOL Plant Working Group. The authors pointed out that further resolution could be achieved with the use of supplementary loci, as non-coding plastid regions, and internal transcribed spacers of nuclear ribosomal DNA, when direct sequencing of this locus is possible. Among the non-coding plastid regions, *trnH-psbA* spacer remains the leading candidate as a source of additional data [8]. In our study, as seen in Table 2, the addition of a third marker did not show significant improvement, raising the identification rate only to 44.44%.

*matK* and *trnH-psbA* showed the highest individual identification rates (Table 2), confirming marker's previously reported species discrimination power. Improvements in *matK* primers' universality and in obtaining high quality *trnH-psbA* bidirectional sequences were not necessary. The suggested *matK* primers performed perfectly for Bromeliaceae. All samples were successfully amplified and sequenced using a single primer pair. Likewise, only two out of 101 samples failed in generating high quality *trnH-psbA* bidirectional sequences after the second trial. Although *rbcL* showed the lowest species identification rate in Bromeliaceae, its sequence variation (overall K2P distance) was similar to the obtained for *trnH-psbA* (Table 2). *matK* sequence variation was twice the amount obtained for the two other markers (Table 2). This trend can also be observed in the pairwise K2P distance distribution frequencies (Figure 1). *matK*/*trnH-psbA* are accounted for the second highest two-locus combination resolution (34.78%), while *rbcL*/*trnH-psbA* almost maintained *trnH-psbA* success identification rate, 26.67% and 26.09%, respectively (Table 2).

In Rosaceae, the combination *rbcL*/*matK* correctly identified 96% of the sampled species [25]. Other families such as Fabaceae [26], Euphorbiaceae [27], and Asteraceae [28] present success identification rates over 80%, using *matK* as a single marker. In our Bromeliaceae data set, even the three regions combined yielded a low identification rate (Table 2).

This species identification failure in Bromeliaceae could also be seen in the analysis of GenBank's *matK* data set. Bromeliaceae exhibited the lowest success rate compared to Asteraceae and Orchidaceae (Table 1). These values seem to be related to their sequence variation (overall K2P distance), since Bromeliaceae sequence divergence was almost three times lower than the observed for the other families.

This low variation rate seen in Bromeliaceae also resulted in poorly resolved tree topologies, regardless of the method that was used to infer the phylogeny. Of the three subfamilies represented in our study, only Tillandsioideae was recovered as monophyletic with a high bootstrap value (98.6%). The monophyly of its two genera, *Tillandsia* and *Vriesia*, was highly supported. None of the other genera from other subfamilies were recovered as monophyletic. In fact, paraphyly has been reported for several Bromeliaceae genera [24]. Species paraphyly was also a common feature in our sampling; only ten were recovered monophyletic (Figure 2). These results suggest that, in the Bromeliaceae, taxon definition below genera may be quite artificial; consequence of a rapid morphological divergence contrasting with a high genetic conservatism.

The lack of barcoding gaps, evidenced by species paraphyly and low identification rates, may be explained by unclear species boundaries. Taxonomy of the group is complicated by interspecific hybridization, a mixture of sexual and asexual reproduction, and recent species divergence, as also seen in wild potatoes [29]. Recent lineages with a great number of species tend to have lots of close relatives, reducing levels of interspecific divergence [30]. Bromeliaceae is an example of recent radiation. Extant subfamilies began to diverge from each other only about 19 Mya [24].

DNA barcoding is an important tool for biodiversity assessment. However, it tends to fail in taxonomy complicated and recently diverged groups, such as Bromeliaceae. The use of sequences from multiple genetic sources may improve this scenario. Next-generation sequencing technologies may provide genomic information for comparisons within and among multiple taxonomic levels [31]. Nevertheless, although limited, generated barcoding data reflects the evolution of the group and should not be ignored.

## Supporting Information

Table S1 Accessions used in the work. All specimens' vouchers are deposited in the herbarium of Rio de Janeiro Botanical Garden (RB).
(DOC)

Table S2 Accession codes of matK sequences downloaded from GenBank, belonging to three angiosperm families.
(XLS)

Table S3 Statistical significance of species discrimination between markers and markers combinations. Significant comparisons are in red (P = 0.05, Tukey test); r = *rbcL*, m = *matK* and t = *trnH-psbA*.
(DOC)
