# Supplementary material for: DNA Barcoding Bromeliaceae: Achievements and Pitfalls
Source: PLoS One. 2012 Jan 5;7(1):e29877. doi: 10.1371/journal.pone.0029877 (PMC3252331; doi:10.1371/journal.pone.0029877)
Supplement: Table S1 — Accessions used in the work. All specimens' vouchers are deposited in the herbarium of Rio de Janeiro Botanical Garden (RB). (DOC) [file pone.0029877.s001.doc]

| **Species name** | **Specimen voucher** | **GenBank Accession Codes** | | |
| --- | --- | --- | --- | --- |
|  |  | ***matK*** | ***rbcL*** | ***trnH-psbA*** |
| *Aechmea aquilega* | Martinelli 15105 | JN202203 | JN202111 | JN204596 |
| *Aechmea bicolor* | Martinelli 15386 | JN202204 | JN202112 | JN204597 |
| *Aechmea bicolor* | Martinelli 15355 | JN202205 | JN202113 | JN204598 |
| *Aechmea blumenavii* | Martinelli 14997 | JN202206 | JN202114 | JN204599 |
| *Aechmea blumenavii* | Martinelli 14966 | JN202207 | JN202115 | JN204600 |
| *Aechmea bromelifolia* | Martinelli 15055 | JN202208 | JN202116 | JN204601 |
| *Aechmea bromelifolia* | Martinelli 15160 | JN202209 | JN202117 | JN204602 |
| *Aechmea bromelifolia* | Martinelli 15243 | JN202210 | JN202118 | JN204603 |
| *Aechmea calyculata* | Martinelli 14905 | JN202211 | JN202119 | JN204604 |
| *Aechmea caudata* | Martinelli 15498 | JN202212 | JN202120 | JN204605 |
| *Aechmea caudata* | Martinelli 14963 | JN202213 | JN202121 | JN204606 |
| *Aechmea caudata* | Martinelli 15033 | JN202214 | JN202122 | JN204607 |
| *Aechmea coelestis* | Martinelli 15733 | JN202215 | JN202123 | JN204608 |
| *Aechmea coelestis* | Martinelli 15814 | JN202216 | JN202124 | JN204609 |
| *Aechmea curranii* | Martinelli 15409 | JN202217 | JN202125 | JN204611 |
| *Aechmea curranii* | Martinelli 15363 | JN202218 | JN202126 | JN204612 |
| *Aechmea curranii* | Martinelli 15433 | JN202219 | JN202127 | JN204610 |
| *Aechmea distichantha* | Martinelli 15245 | JN202220 | - | JN204613 |
| *Aechmea distichantha* | Martinelli 15321 | JN202221 | - | JN204614 |
| *Aechmea lamarchei* | Martinelli 15161 | JN202222 | JN202128 | JN204615 |
| *Aechmea lingulata* | Martinelli 15345 | JN202223 | JN202129 | JN204616 |
| *Aechmea lingulata* | Martinelli 15461 | JN202224 | JN202130 | JN204617 |
| *Aechmea nudicaulis* | Martinelli 15148 | JN202225 | JN202131 | JN204618 |
| *Aechmea nudicaulis* | Martinelli 15013 | JN202226 | JN202132 | JN204619 |
| *Aechmea phanerophlebia* | Martinelli 15155 | JN202227 | JN202133 | JN204620 |
| *Aechmea phanerophlebia* | Martinelli 15752 | JN202228 | JN202134 | JN204621 |
| *Aechmea phanerophlebia* | Martinelli 15325 | JN202229 | JN202135 | JN204622 |
| *Aechmea ramosa* | Martinelli 15159 | JN202230 | JN202136 | JN204623 |
| *Aechmea ramosa* | Martinelli 15143 | JN202231 | JN202137 | JN204624 |
| *Aechmea recurvata* | Martinelli 14935 | JN202232 | JN202138 | JN204625 |
| *Aechmea recurvata* | Martinelli 14879 | JN202233 | JN202139 | JN204626 |
| *Aechmea recurvata* | Martinelli 14893 | JN202234 | JN202140 | JN204627 |
| *Billbergia distachia* | Martinelli 14937 | JN202235 | JN202141 | JN204628 |
| *Billbergia distachia* | Martinelli 15291 | JN202236 | JN202142 | JN204629 |
| *Billbergia euphemiae* | Martinelli 15438 | JN202237 | JN202143 | JN204630 |
| *Billbergia euphemiae* | Martinelli 15470 | JN202238 | JN202144 | JN204631 |
| *Billbergia euphemiae* | Martinelli 15746 | JN202239 | JN202145 | JN204632 |
| *Billbergia saundersii* | Martinelli 15453 | JN202240 | JN202146 | JN204633 |
| *Billbergia saundersii* | Martinelli 15382 | JN202241 | - | - |
| *Billbergia saundersii* | Martinelli 15372 | JN202242 | JN202147 | JN204634 |
| *Billbergia saundersii* | Martinelli 15395 | JN202243 | JN202148 | JN204635 |
| *Canistrum aurantiacum* | Martinelli 15117 | JN202244 | JN202149 | JN204636 |
| *Canistrum aurantiacum* | Martinelli 15102 | JN202245 | JN202150 | JN204637 |
| *Canistrum aurantiacum* | Martinelli 15337 | JN202246 | JN202151 | JN204638 |
| *Hohenbergia ramageana* | Martinelli 15332 | JN202247 | JN202152 | JN204639 |
| *Hohenbergia ramageana* | Martinelli 15115 | JN202248 | JN202153 | JN204640 |
| *Hohenbergia ridleyi* | Martinelli 15343 | JN202249 | JN202154 | JN204641 |
| *Hohenbergia ridleyi* | Martinelli 15346 | JN202250 | JN202155 | JN204642 |
| *Neoregelia laevis* | Martinelli 15009 | JN202251 | JN202156 | JN204643 |
| *Neoregelia laevis* | Martinelli 14911 | JN202252 | JN202157 | JN204644 |
| *Neoregelia pauciflora* | Martinelli 15621 | JN202253 | JN202158 | JN204645 |
| *Nidularium altimontanum* | Martinelli 15479 | JN202254 | JN202159 | JN204646 |
| *Nidularium altimontanum* | Martinelli 15485 | JN202255 | - | JN204647 |
| *Nidularium angustibracteatum* | B.A Moreira 217 | JN202256 | JN202160 | JN204648 |
| *Nidularium angustibracteatum* | B.A Moreira 219 | JN202257 | JN202161 | JN204649 |
| *Nidularium innocentii* | Martinelli 15366 | JN202258 | JN202162 | JN204650 |
| *Nidularium innocentii* | Martinelli 15012 | JN202259 | JN202163 | JN204651 |
| *Nidularium innocentii* | Martinelli 14912 | JN202260 | JN202164 | JN204652 |
| *Nidularium krisgreeniae* | Martinelli 15766 | JN202261 | JN202165 | JN204653 |
| *Nidularium krisgreeniae* | Martinelli 15761 | JN202262 | JN202166 | JN204654 |
| *Nidularium procerum* | B.A Moreira 212 | JN202263 | JN202167 | JN204655 |
| *Nidularium procerum* | Martinelli 14979 | JN202264 | JN202168 | JN204656 |
| *Pitcairnia encholirioides* | Forzza 4158 | JN202265 | - | JN204657 |
| *Pitcairnia encholirioides* | Forzza 3440 | JN202266 | JN202169 | JN204658 |
| *Pitcairnia flammea* | Forzza 2718 | JN202267 | - | JN204659 |
| *Pitcairnia flammea* | Martinelli 15893 | JN202268 | JN202170 | JN204660 |
| *Quesnelia kautskyi* | Martinelli 15667 | JN202269 | JN202171 | JN204661 |
| *Quesnelia kautskyi* | Martinelli 15184 | JN202270 | JN202172 | JN204662 |
| *Tillandsia bulbosa* | Martinelli 15101 | JN202271 | JN202173 | JN204663 |
| *Tillandsia bulbosa* | Martinelli 15457 | JN202272 | JN202174 | JN204664 |
| *Tillandsia geminiflora* | Martinelli 15271 | JN202273 | JN202175 | - |
| *Tillandsia polystachia* | Martinelli 15748 | JN202274 | JN202176 | JN204665 |
| *Tillandsia polystachia* | Martinelli 15151 | JN202275 | JN202177 | JN204666 |
| *Tillandsia tenuifolia* | Martinelli 14914 | JN202276 | JN202178 | JN204667 |
| *Tillandsia tenuifolia* | Martinelli 15256 | JN202277 | JN202179 | JN204668 |
| *Vriesea cacuminis* | Martinelli 15307 | JN202278 | JN202180 | JN204669 |
| *Vriesea cacuminis* | Martinelli 15323 | JN202279 | JN202181 | JN204670 |
| *Vriesea carinata* | Martinelli 15380 | JN202280 | JN202182 | JN204671 |
| *Vriesea carinata* | Martinelli 15723 | JN202281 | JN202183 | JN204672 |
| *Vriesea ensiformis* | Martinelli 15025 | JN202283 | JN202185 | JN204674 |
| *Vriesea erythrodactylon* | Martinelli 15771 | JN202284 | JN202186 | JN204675 |
| *Vriesea erythrodactylon* | Martinelli 15787 | JN202285 | JN202187 | JN204676 |
| *Vriesea erythrodactylon* | Martinelli 14908 | JN202286 | JN202188 | JN204677 |
| *Vriesea friburgensis* | Martinelli 15281 | JN202287 | JN202189 | JN204678 |
| *Vriesea friburgensis* | Martinelli 15310 | JN202288 | JN202190 | JN204679 |
| *Vriesea friburgensis* | Martinelli 15258 | JN202289 | JN202191 | JN204680 |
| *Vriesea friburgensis* | Martinelli 14883 | JN202290 | JN202192 | JN204681 |
| *Vriesea gigantea* | Martinelli 15646 | JN202291 | JN202193 | JN204682 |
| *Vriesea heterostachys* | Martinelli 15505 | JN202292 | JN202194 | JN204683 |
| *Vriesea heterostachys* | Martinelli 15202 | JN202293 | JN202195 | JN204684 |
| *Vriesea incurvata* | Martinelli 14910 | JN202294 | - | JN204685 |
| *Vriesea incurvata* | Martinelli 15765 | JN202295 | JN202196 | JN204686 |
| *Vriesea longicaulis* | Martinelli 15722 | JN202296 | JN202197 | JN204687 |
| *Vriesea longicaulis* | Martinelli 15496 | JN202297 | JN202198 | JN204688 |
| *Vriesea platynema* | Martinelli 15368 | JN202282 | JN202184 | JN204673 |
| *Vriesea platynema* | Martinelli 15822 | JN202298 | - | JN204689 |
| *Vriesea platynema* | Martinelli 15757 | JN202299 | JN202199 | JN204690 |
| *Vriesea procera* | Martinelli 15459 | JN202300 | JN202200 | JN204691 |
| *Vriesea procera* | Martinelli 15556 | JN202301 | JN202201 | JN204692 |
| *Vriesea procera* | Martinelli 15030 | JN202302 | - | JN204693 |
| *Vriesea vagans* | Martinelli 14958 | JN202303 | JN202202 | JN204694 |
