# Supplementary material for: DNA Barcoding Bromeliaceae: Achievements and Pitfalls
Source: PLoS One. 2012 Jan 5;7(1):e29877. doi: 10.1371/journal.pone.0029877 (PMC3252331; doi:10.1371/journal.pone.0029877)
Supplement: Table S3 — Statistical significance of species discrimination between markers and markers combinations. Significant comparisons are in red (P = 0.05, Tukey test); r = rbcL, m = matK and t = trnH-psbA. (DOC) [file pone.0029877.s003.doc]

|  | **r** | **m** | **t** | **r + m** | **r + t** | **m + t** | **r + m + t** |
| --- | --- | --- | --- | --- | --- | --- | --- |
| ***r*** | # |  |  |  |  |  |  |
| ***m*** | 0,1618 | # |  |  |  |  |  |
| ***t*** | 0,2104 | 0,4324 | # |  |  |  |  |
| ***r + m*** | 0,0062 | 0,052 | 0,0408 | # |  |  |  |
| ***r + t*** | 0,1914 | 0,4576 | 0,4712 | 0,0483 | # |  |  |
| ***m + t*** | 0,0446 | 0,2303 | 0,1812 | 0,1974 | 0,2021 | # |  |
| ***r + m + t*** | 0,0051 | 0,0485 | 0,0344 | 0,4634 | 0,0409 | 0,1743 | # |
